# Early Discovery Of Small Bowel Adenocarcinoma In a Patient Admitted For 4 Acute Intestinal Intussusception

**DOI:** 10.1016/j.amsu.2022.104776

**Published:** 2022-09-22

**Authors:** Abdelilah El Bakouri, Anas El Wassi, Yassine Eddaoudi, Mounir Bouali, Khalid El Hattabi, Fatimazahra Bensardi, Abdelaziz Fadil

**Affiliations:** a Visceral Surgery Emergency Department P35 , University Hospital Center Ibn Rochd, Casablanca, Morocco; b Faculty of Medecine and Pharmacy, Hassan II University, Casablanca, Morocco

**Keywords:** Small bowel, Surgery, Adenocarcinoma, Endoscopy, Familial adenomatous polyposis

## Abstract

**Introduction:**

Malignant tumours of the small bowel are uncommon in clinical practice. Adenocarcinoma is the most common of these tumours, accounting for approximately 35–45% of all tumours. It may occur sporadically, in association with familial adenomatous polyposis coli or Peutz-Jeghers syndrome or hereditary non-polyposis colorectal cancer, or in association with chronic inflammatory bowel changes (such as Crohn's disease or celiac disease).

**Materials and methods:**

We report a case of Early Discovery Of Small Bowel Adenocarcinoma In A Patient Admitted For 4 Acute Intestinal Intussusception in the department of Emergency visceral surgery P35 of the ibn rochd hospital in casablanca.

**Results:**

Our patient was admitted to the emergency room for sub-occlusive syndrome with generalized abdominal pain of chronic appearance dating back to one month before his admission With Abdominal and pelvic ultrasound showed: intestinal parietal thickening and minimal ascites (peritoneal and/or intestinal tuberculosis? Crohn's disease)

The patient underwent an abdominal-pelvic CT scan which showed: Presence of diffuse small bowel thickening, involving several small intestines and the colonic angle with intestinal invaginations (at least 3) suspecting an inflammatory or tumoral origin? To be compared with histological data and infiltration of the mesenteric fat in the sub-umbilical region with a peritoneal effusion in the Douglas. the patient was operated on in the emergency room, approached by laparotomy and found on exploration: Presence of 3 invaginations in the small intestine located at 20cm and 90cm from the Duodenojejunal Angle (DIA) as well as at 25cm from the Last part of the small intestine (DAI), with Presence of a colonic invagination at the level of the left colonic angle. the patient underwent 3 small bowel resections and one segmental colonic resection including segmental small bowel resections: the 1st one of 30 cm taking away an invagination of the small intestine at 20cm from the ADJ, the 2nd one taking away 60cm of invaginated located at 90cm from the ADJ the 3rd one taking away 20cm of invaginated located at 25cm from the DAI and a 4th resection taking away an invagination of the left colonic angle with 3 Anastomosis of the T-T small intestine and a transverse Colostomy in Bouilley Volkman.

On examination by the anapathomopathologist: consistent with a small bowel tumour: well-differentiated intestinal adenocarcinoma on degenerated adenomatous polyps measuring 2.5cm and 1.7cm with an estimated 10% mucinous component with no vascular emboli and no peri-nervous sheathing. TNM stage p: pT2 with healthy resection margins in the left colon: Presence of a tubular adenoma with low grade dysplasia.

**Conclusion:**

The most common symptoms of adenocarcinoma of the small bowel are obstruction, overt or covert bleeding, weight loss and jaundice. Because the small bowel has long been relatively inaccessible to routine endoscopy, the diagnosis of small bowel adenocarcinoma was often delayed for several months after the onset of symptoms. Therefore, in case of suspicion of this type of cancer, a thorough evaluation should be undertaken. Nowadays, endoscopy of the small bowel is widely available, allowing an earlier non-invasive diagnosis.

## Highlights

• Acute Intestinal Intussusception as a cause of intestinal obstruction is often a diagnostic challenge mimicking a wide spectrum of diseases.
• Malignant tumours of the small bowel are uncommon in clinical practice. Adenocarcinoma is the most common of these tumours.
• Its diagnosis is still very difficult.
• The treatment of Acute Intestinal Intussusception is in most cases surgical.
• The diagnosis of Acute Intestinal Intussusception is histological.

## 1 Introduction

Although the small intestine accounts for 75% of the length of the gastrointestinal tract and 90% of its mucosal surface area, small bowel adenocarcinomas (SBIs) are rare cancers accounting for less than 2% of all digestive tumours [1]. Adenocarcinomas and endocrine tumours are the main malignant tumours of the small intestine (36.9 and 37.4%, respectively), followed by lymphomas and stromal tumours [2].

Small bowel adenocarcinomas account for approximately 40% of small bowel cancers [1,2]. Small bowel adenocarcinomas are 50 times less common than colon adenocarcinomas [1].

Epidemiological data suggest that the annual incidence of small bowel adenocarcinoma is 2.2–5.7 per million population per year in developed countries [3]. The median age of onset is in the sixth decade [3]. The duodenum is the most frequently affected segment accounting for 55–82% of cases followed by the jejunum (11–25%) and ileum (7–17%) [1]. The increase in the incidence of GIA seems to be mainly due to the increased incidence of duodenal tumours [4,5].

The rarity of the condition makes epidemiological studies difficult, and it has been suggested that consumption of alcohol, tobacco, carbohydrates, red meat and smoked foods are associated with an increased risk of small bowel adenocarcinoma, whereas a reduced risk was observed in consumers of coffee, fish, fruit and vegetables [1].

The work has been reported in line with the SCARE criteria [6].

## 2 Patient and observation

This is a 28-year-old patient admitted to the emergency room for sub-occlusive syndrome with generalized abdominal pain of chronic appearance dating back to one month before his admission with clinical examination: The patient was conscious, hemodynamically and respiratorily stable. The examination revealed generalized abdominal tenderness with slight abdominal distension, the hernial orifices were free. The biological work-up revealed a hb 12.4 g/dL; hyperleukocytosis with a predominance of PNNs at 18,300 elements/mm3, the CRP was high at 215mg/L, the renal function was normal, urea 5 mmol/L, creatinemia 9 mg/L. Abdominal and pelvic ultrasound showed: intestinal parietal thickening and minimal ascites (peritoneal and/or intestinal tuberculosis? Crohn's disease).

**Fig. 1:**
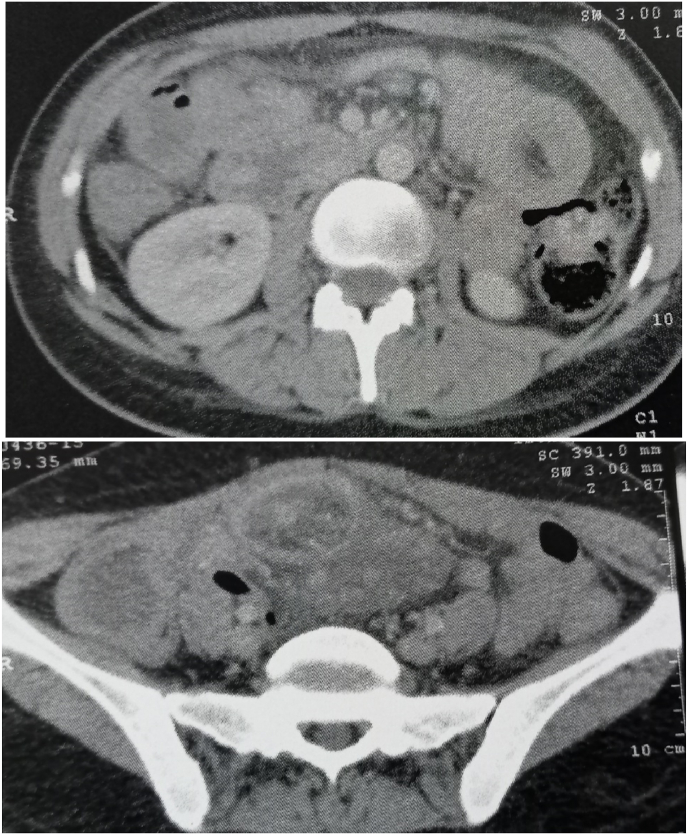
abdominal-pelvic CT.

The patient underwent an abdominal-pelvic CT scan which showed: Presence of diffuse small bowel thickening, involving several small intestines and the colonic angle with intestinal invaginations (at least 3) suspecting an inflammatory or tumoral origin? To be compared with histological data and infiltration of the mesenteric fat in the subumbilical region with a peritoneal effusion in the Douglas (Fig. 1).

**Fig. 2:**
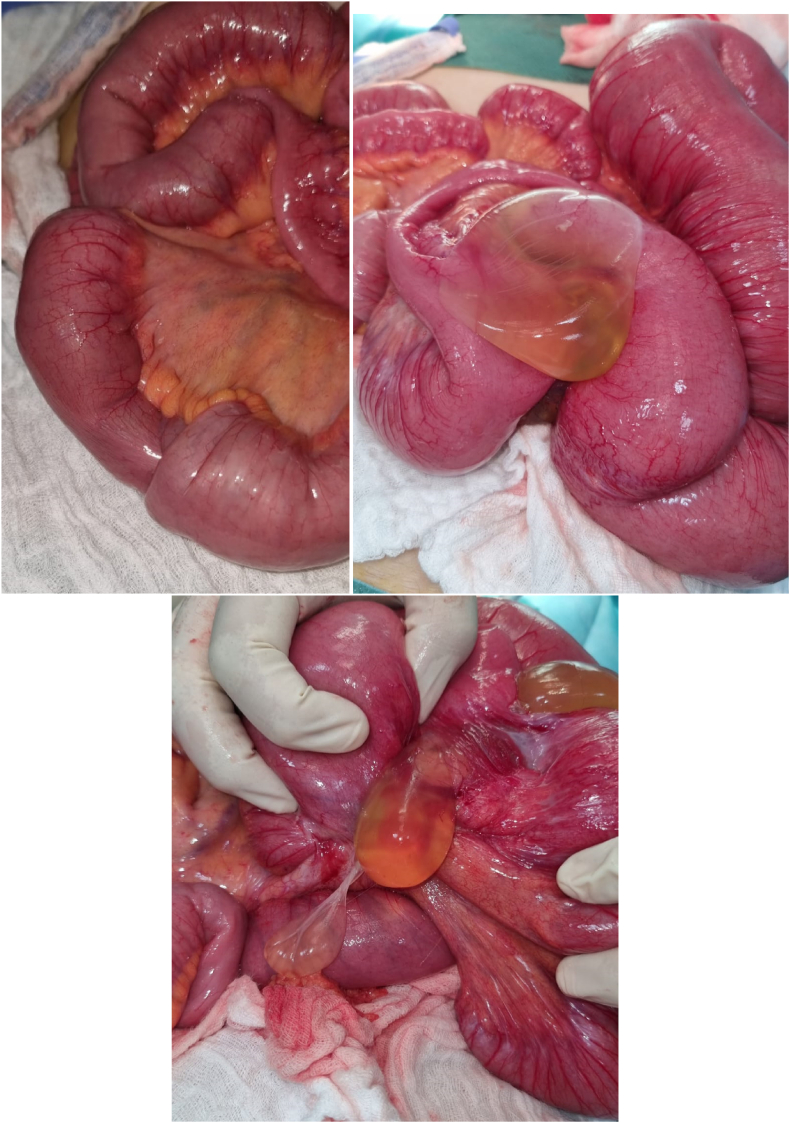
Intraoperative view showing of 3 invaginations in the small intestine.

**Fig. 3:**
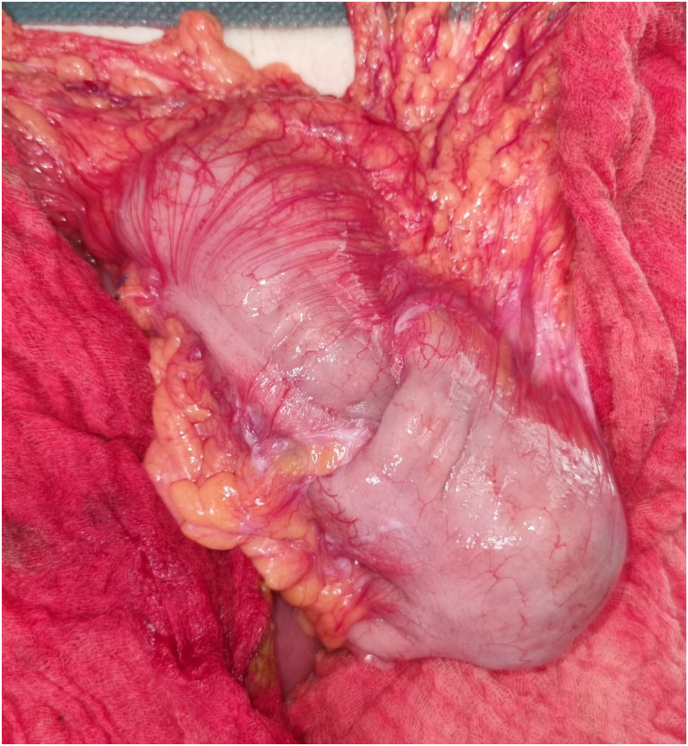
Intraoperative view showing:colonic invagination at the level of the left colonic angle.

The patient was operated on in the emergency room in the department of Emergency visceral surgery P35 of the ibn rochd hospital in casablanca, approached by laparotomy and found on exploration: Presence of 3 invaginations in the small intestine located at 20cm and 90cm from the Duodenojejunal Angle (DIA) as well as at 25cm from the Last part of the small intestine (DAI) (Fig. 2), with Presence of a colonic invagination at the level of the left colonic angle (Fig. 3).

**Fig. 4:**
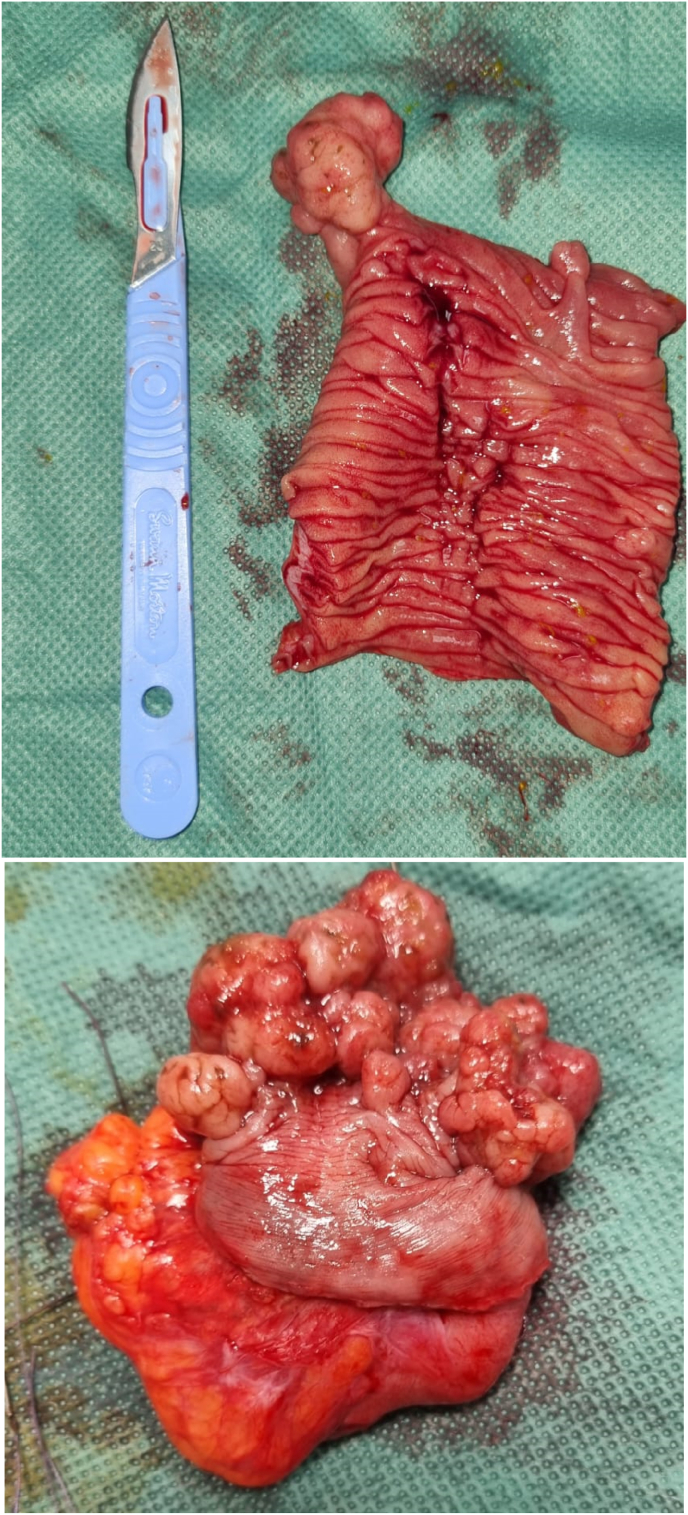
After operative view showing.

The patient underwent 3 small bowel resections (Fig. 4) and one segmental colonic resection including segmental small bowel resections: the 1st one of 30 cm taking away an invagination of the small intestine at 20cm from the ADJ, the 2nd one taking away 60cm of invaginated located at 90cm from the ADJ the 3rd one taking away 20cm of invaginated located at 25cm from the DAI and a 4th resection taking away an invagination of the left colonic angle with 3 Anastomosis of the T-T small intestine and a transverse Colostomy in Bouilley Volkman.

On examination by the anapathomopathologist: consistent with a small bowel tumour: well-differentiated intestinal adenocarcinoma on degenerated adenomatous polyps measuring 2.5cm and 1.7cm with an estimated 10% mucinous component with no vascular emboli and no peri-nervous sheathing. TNM stage p: pT2 with healthy resection margins in the left colon: Presence of a tubular adenoma with low grade dysplasia.

The surgical procedure was performed on a scheduled date with a correct pre-anesthetic assessment; the procedure was performed by an assistant professor in general surgery and two residents in the same specialty.

The operation was performed in the operating room of the P35 visceral emergency department at the CHU ibn rochd hospital in Casablanca, Morocco

The patient was satisfied with the intervention and the improvement of his health in the short and long term.

## 3 Discussion

The most important risk factor for adenocarcinoma of the small bowel is the existence of a pre-existing adenoma, either isolated or multiple in association with one of the multiple polyposis syndromes [3]. Adenocarcinomas, particularly those of the duodenum, become symptomatic long before other small bowel tumours, allowing early detection and therapeutic intervention. However, most carcinomas of the small intestine are discovered at a metastatic stage [4].

Small bowel adenocarcinomas are usually treated as colon cancers, with curative treatment being surgical. Adjuvant chemotherapy is often proposed as in colon cancer depending on risk factors. Overall survival at 5 years is 30.5%, with a median of almost 2 years [5].

Knowledge of the natural history of these cancers is limited and this is due both to their rarity and to the variety of different tumour types encountered [7]. The most common symptoms of small bowel adenocarcinoma are chronic abdominal pain (60%), overt or occult bleeding (50%), anaemia (50%), obstruction (30%), and altered general condition [8]. Certain hereditary syndromes, certain inflammatory alterations of the small intestine are associated with an increased incidence of small intestine adenocarcinoma such as familial adenomatous polyposis [9], juvenile familial polyposis, sporadic adenomas, Crohn's disease [10], dietary factors such as tea, sugar, coffee or aromatic amino acids could also predispose to the development of small intestine adenocarcinomas [11].

Familial adenomatous polyposis (FAP), linked to a mutation in the APC gene, is characterised by

Multiple adenomatous polyps in the colon and rectum that sooner or later progress to carcinoma. The vast majority of these patients also develop duodenal polyps that degenerate into adenocarcinoma in 4% of cases [[12], [13], [14], [15]]. In a series of 1255 patients with FAP, 4.5% had developed upper GI adenocarcinoma. Of these patients, 51% had adenocarcinoma in the duodenum, 17% in the ampulla of Vater and 12% in the stomach. The risk of developing adenocarcinoma of the jejunum or ileum for these patients was not significant [13,16,17].

Routine screening for duodenal and ampullary polyps is recommended in patients with FAP. The Spigelman et al. [12] classification of duodenal polyps is used to define the surveillance strategy. This classification is based on the number, size, histological type.

The classification is based on the number, size, histological type and degree of dysplasia of the polyps, distinguishing between minor (stage I/II) and major (stage III/IV) polyps, the latter being four times more likely to degenerate [13,18]. Monitoring of these patients is based on endoscopy every three to four years for stage I or II and annual endoscopy with biopsies for stage III or IV [19]. In some forms of severe polyposis with a major risk of degeneration, surgical duodenopancreatectomy with or without preservation of the pylorus may be justified to prevent the development of duodenal cancers [18,20,21].

Adenocarcinomas are infrequently found in clinical practice, due to the absence of specific clinical signs, and often it is the emergency laparotomy imposed by an acute event that allows them to be discovered, which is the same as in our patient, or they are already diagnosed at the metastasis stage. Diagnosis can be particularly difficult for ileal adenocarcinomas when they occur in the context of Crohn's disease because the tumour clinically simulates a recrudescence of the inflammatory process of the disease or a fibrous stenosis [22].

Approximately one third of patients have distant metastases (stage IV) and one third have lymph node involvement (stage III) at diagnosis [23,24].

Until recently, the main tests available to clinicians to explore the small bowel were abdominopelvic CT and small bowel transit [25,26]. Small bowel transit can demonstrate a small bowel tumour in the form of an annular apple core stenosis or a short, irregular segment of the intestinal mucosa. Abdominal CT may show a steeply sloping annular stricture with circumferential parietal thickening enhanced by contrast injection. New diagnostic methods such as enteroscanner or entero-MRI, videocapsule and enteroscopy currently allow better exploration of the small intestine and should allow earlier diagnoses, particularly in the case of anaemia due to unexplained martial deficiency.

Enteroscanner is a technique for exploring the small intestine that combines the advantages of two known techniques, enteroclysis and multidetector CT. It consists of filling the small intestine with water through a naso-jejunal tube, followed by a spiral CT scan in thin sections. This method is easily performed and is more informative than small bowel transit, and can guide enteroscopy and endoscopic videocapsule (EVC). Enteroscopy allows the detection of small bowel tumours with a sensitivity of 85–95% and a specificity of 90–96%, and at the same time allows the assessment of tumour extension with the search for distant metastatic localisation [[27], [28], [29], [30]].

Entero-MRI is a promising diagnostic imaging examination with the advantage of not irradiating patients. The image acquisition modalities are based on the same small bowel distension techniques as the enteroscanner. The sensitivity and specificity of entero-MRI for the detection of small bowel tumours are 86 and 98%, respectively [[31], [32], [33]].

Surgery is the treatment of choice and the only potentially curative treatment modality. Duodenal tumours may require cephaloduodenopancreatectomy; in other locations, simple resection of the affected segment and mesentery with lymph node dissection. Small adenocarcinomas, especially polyploid lesions with limited mucosal or submucosal infiltration, can sometimes be treated by endoscopic resection [34]. Currently, there is no consensus on the use of adjuvant chemotherapy after curative surgical treatment. The various studies conducted on the use of adjuvant chemotherapy in small adenocarcinoma were designed for groups of patients at high risk of recurrence, considered to have a poor prognosis compared with patients who do not receive adjuvant chemotherapy. Most centres use 5FU-based mono-chemotherapy [5]. The role of radiotherapy as a component of adjuvant treatment for duodenal adenocarcinoma has been studied to a limited extent, in a series from Duke University, no difference in overall 5-year survival was observed between patients who did and did not receive radiotherapy in combination with adjuvant or neoadjuvant 5FU. However, in the subgroup of patients with a negative resection margin, the 5-year overall survival was 53% in the surgery alone group and 83% in the chemoradiotherapy group [35]. In the metastatic setting, two prospective studies have been conducted: a phase II study (CAPOX) evaluated the benefit of the combination of capecitabine and oxaliplatin in 30 patients with metastatic or locally advanced small bowel adenocarcinoma: The overall response rate was 50%, with a median time to progression of 11.3 months and a median overall survival of 20.4 months. The overall response rate was 50%, with a median time to progression of 11.3 months and a median overall survival of 20.4 months [19], when administered to patients with good performance status, CAPOX is well tolerated with a superior response rate in terms of overall survival compared to other regimens in the literature [36]. CAPOX should be considered as a new standard for advanced small cell carcinoma [5]. The role of targeted therapies, including antiangiogenic (anti-VEGF) and epidermal growth factor inhibitors (anti-EGFR), has not yet been evaluated in small cell carcinoma [5], A multicentre study conducted by the Eastern Cooperative Oncology Group (ECOG) reported on the combination of 5-FU, doxorubicin and mitomycin C (MCM) in 39 patients with adenocarcinoma of the small bowel or ampulla of Vater. The overall response rate was 18%, with a median overall survival of 8 months [37].

The prognosis of small bowel adenocarcinoma can be improved by The age of the patient, the location of the tumour, its stage and the possibility of performing radical oncological surgery represent the factors related to survival. The 5-year overall survival is 30.5%, with a median of 19.7 months [38,39].

## 4 Conclusion

Adenocarcinomas of the small intestine pose diagnostic problems because of their rarity and the absence of specific clinical signs. They are often discovered surgically during an acute complication. The basic treatment is surgical, the benefit of adjuvant chemotherapy is not well established due to the scarcity of prospective and retrospective studies. The diagnosis of tumours located in other segments of the small bowel is typically made either by double-contrast small bowel transit or most often by enteroscopy [40,41]. The presence of metastases can be assessed by thoracic-abdominal-pelvic CT. Their long-term prognosis is still poor and may improve with additional treatments, hence the need for further studies and inclusion of patients in clinical trials.

## Ethical approval

As per international standard written ethical approval has been collected and preserved by the author(s).

## Sources of funding

None.

## Author contribution

This work was carried out in collaboration among all authors. All authors contributed to theconduct of this work. They also declare that they have read and approved the final version ofthe manuscript.

## Research registration

None.

## Guarantor

Dr ELW ED.

## Consent

Written informed consent was obtained from the patient for publication of this case report and accompanying images. A copy of the written consent is available for review by the Editor-in-Chief of this journal on request.

## Provenance and peer review

Not commissioned, externally peer-reviewed.

## Declaration of competing interest

None.
